# Deep learning hybridization for improved malware detection in smart Internet of Things

**DOI:** 10.1038/s41598-024-57864-8

**Published:** 2024-04-03

**Authors:** Abdulwahab Ali Almazroi, Nasir Ayub

**Affiliations:** 1 Department of Information Technology, College of Computing and Information Technology at Khulais, University of Jeddah, Jeddah, 21959 Saudi Arabia; 2 Department of Creative Technologies, Air University Islamabad, Islamabad, 44000 Pakistan

**Keywords:** IoT security, Malware detection, Artificial intelligence, BERT-based neural network, Optimization, Computational methods, Computational science, Computer science, Scientific data

## Abstract

The rapid expansion of AI-enabled Internet of Things (IoT) devices presents significant security challenges, impacting both privacy and organizational resources. The dynamic increase in big data generated by IoT devices poses a persistent problem, particularly in making decisions based on the continuously growing data. To address this challenge in a dynamic environment, this study introduces a specialized BERT-based Feed Forward Neural Network Framework (BEFNet) designed for IoT scenarios. In this evaluation, a novel framework with distinct modules is employed for a thorough analysis of 8 datasets, each representing a different type of malware. BEFSONet is optimized using the Spotted Hyena Optimizer (SO), highlighting its adaptability to diverse shapes of malware data. Thorough exploratory analyses and comparative evaluations underscore BEFSONet’s exceptional performance metrics, achieving 97.99% accuracy, 97.96 Matthews Correlation Coefficient, 97% F1-Score, 98.37% Area under the ROC Curve(AUC-ROC), and 95.89 Cohen’s Kappa. This research positions BEFSONet as a robust defense mechanism in the era of IoT security, offering an effective solution to evolving challenges in dynamic decision-making environments.

## Introduction

Modern technologies such as big data, 5G, computational intelligence, and the Internet of Things (IoT) are converging to reshape various sectors as we navigate the uncertainties of the Fourth Industrial Revolution^1^. The synergy, especially between IoT, AI, and 5G, is propelling the integration of smart technologies into diverse industries like smart automobiles, factories, and cities^2^. While this transformation is revolutionizing industries, the growing IoT market is not only reshaping industrial landscapes but also influencing our daily lives in the long term. However, the interconnectivity of IoT devices exposes them to an increasing array of cyber threats, including botnet operations, cryptocurrency mining, and distributed denial-of-service (DDoS) attacks^3^. Mass manufacturing to meet the demand for IoT devices introduces vulnerabilities, elevating the risk of security breaches^4^. Insecure IoT devices pose threats to user information and can infiltrate large networks, accelerating the spread of malware^5^.

The urgency of protecting IoT nodes becomes evident in the face of escalating security risks, such as the Mirai virus, which has orchestrated catastrophic DDoS attacks since October 2021^6^. Mirai exploits vulnerabilities in IoT devices, negatively impacting device efficiency and throughput through hostile botnets^7^. Such attacks raise financial and productivity concerns for global corporations, underscoring the need for comprehensive solutions. With the continued growth of IoT devices, the potential for large-scale attacks like DDoS grows, emphasizing the critical importance of device security and malware prevention. The Mirai virus serves as a stark reminder of the crucial significance of these aspects. Inadequate awareness and inefficient maintenance contribute to the complexity of malware variations, making effective mitigation challenging^8^.

The fundamental principle of transforming physical entities into virtual entities, inherent in the Internet of Things, extends to various aspects of our lives, including healthcare, smart homes, agriculture, and industry. The Industrial IoT (IIoT) significantly improves efficiency and overall performance by establishing critical links between supply chains, manufacturing processes, and end-users^9^. However, this interconnected ecosystem also introduces security challenges, leading to the initiation of research efforts focused on IoT malware detection through feature learning and classification^10^. Despite various detection approaches, IoT devices face challenges due to limited hardware resources specialized for specific capabilities. Intelligent and rapidly evolving IoT malware poses a challenge, as does evaluating the vast behavioral data created by IoT malware^11^. To address these challenges, Machine Learning (ML) emerges as a potential solution, offering a comprehensive approach to data preparation, evaluation, and cross-validation using algorithm-based learning curves^12^.

ML approaches, particularly those employing Deep Learning (DL) and advanced convolutional neural networks (CNNs), have demonstrated effectiveness in examining flaws in IoT firmware and apps, showcasing their ability to recognize and classify different kinds of IoT malware^13^. Deep CNNs, in particular, excel in deciphering complex aspects of IIoT malware by extracting discriminative properties at various abstraction levels^13^. As research trends favor neural networks, especially deep CNNs, their ability to decode intricate features within the IoT malware domain becomes increasingly evident. In this context, the introduction of AI, particularly ML and DL, stands as a promising avenue for addressing the security challenges faced by IoT devices^14–16^. These approaches have shown robustness in enhancing anti-malware programs and adapting to the evolving nature of IoT threats. By leveraging AI, we aim to provide a comprehensive and adaptive solution to fortify IoT security in the face of dynamic and sophisticated cyber threats.

A novel ensemble method for malware identification and categorization on IoT devices is presented in this paper. The proposed model, called BEFSONet, integrates BERT with an optimization technique called Spotted Hyena Optimizer (SHO) and a feed forward neural network called BEFSONet. This results in a highly accurate and complex malware identification and categorization process. Mean Decrease Impurity (MDI) patterns are used to extract significant features from malware samples, which are subsequently put into ML models for additional feature extraction and analysis. SHO improves the performance of the model by determining optimal parameter choices and lowering computing complexity. This study’s main contributions include:

1. Comprehensive Dataset Examination: To properly train the model, this study meticulously examined eight important IOT23 datasets containing malware and benign occurrences, emphasizing the foundational step towards advancing robust IoT security solutions.
2. Malware Behavior Identification: The model identifies sophisticated malware behaviors through adept feature crafting methodologies, including meticulous feature normalization, extensive categorical encoding, and insightful analysis of feature significance using Mean Decrease Accuracy (MDA) and Random Forest (RF). This capability is crucial for enhancing the sophistication of IoT security solutions.
3. Innovative DL Ensemble (BEFSONetO): A novel DL ensemble, BEFSONetO, was specifically designed to effectively evaluate and categorize large datasets, showcasing advancements that contribute significantly to the efficiency and efficacy of IoT security solutions.
4. Improved Classification Performance: The suggested method, BEFSONet, achieves a noteworthy 17% improvement in classification performance accuracy, coupled with a 12% reduction in time complexity compared to existing methodologies. These enhancements underscore the practical impact of the research on refining IoT security solutions.
5. Efficient Model Parameter Optimization: By leveraging SHO to optimize the BEFSONet parameters, the model exhibits efficient handling of extensive datasets with consistent accuracy and computational speed, marking a substantial contribution to the optimization of IoT security solutions.
6. Practical Applicability: Eight distinct malware strains were successfully identified and protected against, significantly enhancing the security of IoT devices. This practical applicability highlights the immediate and tangible impact of the research on strengthening IoT security solutions, especially in critical sectors like smart cities and contemporary manufacturing.
7. Resource Efficiency: This research enhances resource efficiency through the exploration of lightweight model architectures and efficient algorithms in the proposed ensemble model (BEFSONet). This focus on resource efficiency is pivotal for the practical implementation of IoT security solutions on resource-constrained devices.
8. Scalability Considerations: Addressing scalability challenges in IoT environments, this study employs strategies like distributed computing and parallel processing to ensure the adaptability and optimal performance of the proposed ensemble model (BEFSONet). This scalability focus is instrumental in accommodating the growing volume of IoT devices and data, further contributing to the advancement of IoT security solutions.

The following is the order of the sections of the article: Section “Related work” provides a brief summary of previous studies on malware categorization and detection. Section “Proposed system model” explores the basic parts and workings of the modified hybrid model in further detail. The experimental findings are also summarized in section “Simulation and results”, where the effectiveness of the recommended ensemble is assessed. Section “Conclusion and future directions” concludes up the analysis with a review of the key discoveries and recommendations for further lines of inquiry.

### Motivation

The field of malware detection has changed, incorporating advanced data methodologies, ML, ensemble techniques, static and dynamic analysis, and other features^12,13,17–20^. However innovative approaches are essential, especially given artificial intelligence’s increasing impact in the field of malware detection. The training and selective use of several classifiers in ensemble learning is a viable approach to improve detection skills. Even while signature-based methods are widely used in detection, they still have limitations, mainly in terms of handling known malware variants. Moreover, it is frequently time-consuming to integrate passive and active analytic methodologies. The problem of disparity in class between normal and malignant instances has been well reported in the literature; yet, it remains a persistent challenge that calls for sophisticated solutions to properly resolve^21–24^. To examine the runtime behavior of malware, this work presents a novel technique that combines static and dynamic analysis in light of these difficulties. The substantial development of combining ensemble learning with the SHO optimization technique and the BEFSONet classifier may lead to increased efficiency and efficacy in malware detection.

## Related work

Several research endeavors have delved into the intricacies of malware analysis, employing diverse techniques to comprehend the inner workings and functionality of malware before its detection. While static analysis offers a comprehensive understanding of malware structure without execution, its limitations become evident when deciphering general malware functionalities and uncovering obfuscated malware through packing^17–20^.

In response to the challenges posed by static analysis, dynamic analysis techniques have gained popularity. These techniques aim to assess the overall functionality of malware, identify new and variant malware during execution, and detect disguised malware^21^. Another way of exploration involves the conversion of significant feature data produced by malware into images for enhanced malware detection^25^. The Automated and Behavior-based Malware Assessment and Labelling (AMAL) system, presented by^26^, comprises MaLabel and AutoMal. MaLabel establishes family-based malware classifications, employing ML techniques such as SVM, Trees Algorithm, and Clustering algorithms. However, the manual verification of AMAL by malware analysts introduces subjectivity in selecting and classifying representative malware behaviors. Utilizing API which regularly to capture calls and arguments, the author in^27^ introduces a behavior analysis technique. ML techniques, including Random Forest (RF), SVM algorithms, and DT, are employed for classification, deducing unique malware behaviors from the resulting API sequence. Nevertheless, the subjective involvement of analysts in understanding malware behaviors remains a challenge.

An economically viable host-centered botnet detection method is presented by^22^, demonstrating cost-effectiveness but with the drawback of computational overhead. In^23^, auto-encoders are employed, achieving good accuracy, especially with abundant true positive data, without explicit mention of restrictions.

**Table 1:** Summary of the existing methodologies.

References	Methodology uses	Advantages/pros	Limitations
^17–20^	Static analysis	Offers in-depth insight into malware structure without execution	Struggles in deciphering general malware functionalities and revealing obfuscated malware through packing
^21^	DenseNet	Assesses overall malware functionality, identifies new and variant malware during execution, and detects disguised malware	N/A
^25^	Conversion of feature data into images	Enhances malware detection through visual representation	N/A
^26^	AMAL system with MaLabel and AutoMal	Establishes family-based malware classifications using SVM, DT, and KNN algorithms	Manual verification introduces subjectivity in selecting and classifying representative malware behaviors
^27^	Behavior analysis using API calls and arguments	Classifies malware using RF, SVM, and DT, inferring unique malware behaviors from API sequences	Subjective involvement of analysts in understanding malware behaviors
^22^	Host-centered botnet detection	Demonstrates cost-effectiveness	Introduces computational overhead
^23^	Auto-encoders, LSTM	Achieves good accuracy, especially with abundant true positive data	timeline not defined
^24^	SVM for malware identification	Boasts accuracy but faces issues with time consumption and missing data	Model overfitted on large data
^28^	SVM compatibility with IDS	Maintains a low false alarm rate	Vulnerable to attacks due to intrinsic flaws and potential delays in packet arrival
^29^	Kernel-based learning	Showcases excellent accuracy and resilience	Computational complexity remains undisclosed
^30^	IoBT	Demonstrates remarkable accuracy and precision	Raises concerns about suitability for self-driving cars
^31^	Fusion properties	Tailored for autonomous driving scenarios	Computational inefficient
^32^	Blockchain-based strategy	Offers affordability but proves incompatible with IDS	N/A
^33^	Blockchain technology	Introduces increased processing time	Inefficient interms of Time Complexity and Space
^34^	ResNet	Demonstrates accuracy in identifying zero-day attacks	Faces challenges in effectively handling traffic difficulties
^32^	SVELTE technique	Achieves minimal computing costs	Struggles to handle traffic difficulties effectively
^35^	Signature-based analysis	Effectively mitigates computational overhead	Encounters a high False Negative Rate (FNR)
^34^	CNN	Yields a low FNR	Vulnerable to complicated code encryption
^36^	SVM implementation	Ensures accuracy but introduces computation overhead	model overfitting
^37^	DenseNet	Boasts a high detection rate but reveals security flaws	inefficient for diverse dataset
^38^	Naïve Bayes method	Ensures accuracy and resilience	regularization biased
^39^	GhostNet-GRU	Yields excellent results for malware detection	reduced capacity for handling complex and diverse datasets
^40^	CNN	Maintains minimal processing expense	Unsuitable for complex designs
^41^	CNN	Achieves an accuracy increase without detailing computational complexity	Computational complexity not detailed
^42^	CNN	Produces commendable results but encounters difficulties due to a high FNR	Faces a high False Negative rate

Employing Support Vector Machines (SVM) for botnet identification^24^, identifies accuracy as a strength but notes issues with time consumption and missing data. SVM is also used by^28^, highlighting compatibility with Intrusion Detection Systems (IDS) and a low false alarm rate. However, vulnerabilities to assaults due to intrinsic flaws are acknowledged, alongside potential delays in packet arrival. HaddadPajouh^29^ introduces a kernel-based learning technique, showcasing excellent accuracy and resilience without detailing the computational complexity of the model.

While introducing the IoBT and demonstrating its remarkable accuracy and precision^30^, expresses concerns about its suitability for use in the context of self-driving cars. Fusion properties presented by^31^ are particularly well-suited for autonomous driving. A scalable blockchain-based strategy attempted by^32^ showcases affordability but proves incompatible with IDS.^33^ adopts blockchain technology similarly, albeit at the cost of increased processing time.

Static analysis by^34^, though facing challenges in identifying zero-day attacks, demonstrates accuracy. The SVELTE technique of^32^, while achieving minimal computing costs, struggles to handle traffic difficulties effectively^35^. tackles the issue of a high False Negative Rate (FNR) with signature-based analysis, mitigating computational overhead^34^, utilizing Convolutional Neural Networks (CNN), achieves a low FNR but is susceptible to complicated code encryption.

Using a visualization method^36^, effectively detects zero-day attacks without known constraints. Although^23^ guarantees accuracy with SVM implementation, computation overhead is experienced. Hemalatha et al.^37^ presents a black-box technique, boasting a high detection rate but revealing security flaws.

The Naïve Bayes method by^38^ ensures accuracy and resilience without imposing specific restrictions. A statistical study by^39^ yields excellent results but operates at peak efficiency solely on Windows OS. Li et al.^40^, employing CNN with minimal processing expense, acknowledges its unsuitability for complex designs. Abdullah et al.^41^, also utilizing CNN, observes an accuracy increase without detailing the computational complexity. Finally, the CNN method by^42^ produces commendable results but encounters difficulties due to a high False Negative rate. Table 1 displays the condensed presentation of the related work.

## Proposed system model

**Figure 1:**
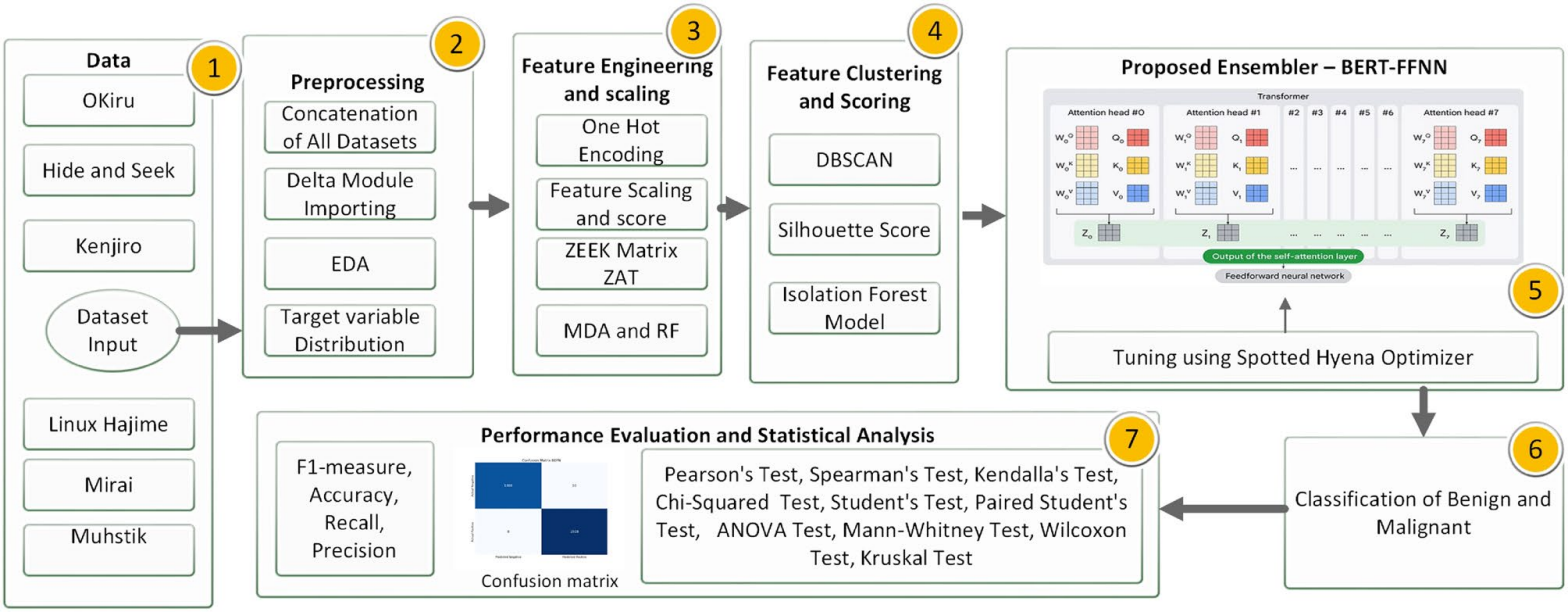
Proposed BEFSONet malware detection model.

This paper presents a hybrid deep-learning approach to malware detection that employs the use of a diverse set of eight unique malware datasets. Figure 1 shows a visual illustration of the process of the proposed paradigm.

In the first step, the datasets are loaded into data frames and combined according to the target column. This allows for the separation of harmful and benign occurrences for further analysis using graphical data analysis. We take mreasures to mitigate overfitting by thoroughly analyzing the spread of the target variable, recognizing that imbalances in datasets could lead to challenges. Following that, The input data is converted into a structure that can be processed by DL using Single Hot Encode and Feature Engineering. Next, using feature scaling, the data is normalized and aligned with the typical range of independent variables. When dealing with vast volumes of data, the Zeek Analysis Tool (ZAT) data frame is useful since it contains essential aspects that are found by using the Mean of Reduction in Accuracy (MDA) and RF techniques. The DBSCAN method is used to cluster the information, and the silhouette rating metric is used to evaluate the effectiveness of various clustering strategies. The isolation tree approach is used to find any differences or abnormalities in the dataset after the clustering phase. After completing these preparation processes, DL analysis is performed on the dataset. Before classification, we divided the data into two parts: 80 percent is test data, and 20 percent is training data. The SHO optimization method is used to optimize BEFSONet parameters, which are essential for improving classification.

### Dataset collection and description

IoT 23 is a significant resource that was created particularly to gather network packets flow from the IoT devices. Twenty distinct scenarios are included in this dataset, which include both instances of malware-induced IoT device compromise and benign IoT traffic^43^. These scenarios are methodically organized and include three legitimate network traffic samples from common IoT devices, as well as an additional twenty pcap files depicting scenarios involving compromised devices. Particularly, the pcap files are modified every 24 hours, a metric ascribed to the malware’ dynamic nature and the significant traffic created during each transmission. However, it is important to emphasize that several pcap files had to be stopped for over a day owing to their large size, resulting in variances in capture lengths.

**Table 2:** Summarized view of IoT dataset.

S. no.	Name of dataset	Name	Duration (h)	Packets	Pcap size (MB)	Zeek flows
1	IOT-2-CTU	Hide and Seek	24	1,787,000	174	1,008,751
2	IOT-61-CTU	Gagfyt	13	220,000	2.9	35,812
3	IOT-18-CTU	Kenjiro	23	52,000	434	54,660
4	IOT-49-CTU	Mirai	23	133,000	1.35	3,394,347
5	IOT-10-CTU	Linux Hajime	23	638,000	8.34	6,378,295
6	IOT-4-CTU	Muhstik	35	499,000	55	156,105
7	IOT-5-CTU	Tori	23	54,000	4.0	3,289
8	IOT-4-CTU	Okiru	23	1,302,000	21	1,364,514

Table 2 contains detailed information on the 20 scenarios, including ID_scenario, name of dataset, length(hours), transaction count, ID_Zeek, and information collected from the conn.log document using Zeek’s network analysis framework. The table also provides details regarding the size of the pcap files as well as probable names connected with the variants of malware used to infect each device. The IoT 23 dataset is valuable because it has the potential to assist researchers and developers working on security projects by offering a platform for improving detection models and machine-learning algorithms. With its wide range of network traffic situations, it is an invaluable tool for education, helping with the teaching and assessment of security solutions.

Stratosphere laboratories carefully craft the labels in the IoT-23 dataset through a detailed examination of malware captures, which allows them to describe connections that are linked to illegal or potentially hazardous activity. These labels are important identifiers of malicious traffic, typically revealing patterns of activity^43^. The term “attack” describes efforts to take advantage of weak services on hosts other than the compromised device. Flows that show attempts to take advantage of weak services, such as brute force telnet login attempts and GET request header command injection assaults, are included in this category. Conversely, connections with no questionable or dangerous behavior are indicated by the Benign designation. Control and Communication (C &C) servers are connected to devices with the C &C designation. Frequent visits to fraudulent websites, file downloaded files, or the discovery of encoded or IRC-like instructions are indicators of this activity. The Distributed Denial of Service (DDoS) label indicates that the compromised device is launching a DDoS assault, sending several data streams to a single IP address.

FileDownload refers to connections where files are downloaded to devices that have been hacked. Identifying these associations entails closely examining communications with address bytes greater than 3KB or 5KB, frequently focusing on certain dubious ports for destinations or IPs connected to C &C servers. Connections that are identified by the HeartBeat tag enable the C &C server to keep an eye on the compromised host. These connections are often identifiable and associated with response data smaller than one kilobyte (KB). Connections that meet the requirements for a Mirai botnet-a common attack vector-are designated with the Mirai label. Similar to Mirai, but with lower frequency, the Okiru label indicates connections suggestive of an Okiru botnet. The parameters for classification are comparable to those employed in the case of Mirai.

Horizontal port scans are used to get information about impending attacks on connections identified with the PartOfAHorizontal-PortScan label. Recognizing patterns where connections share a range of IP addresses, utilize the same port, and transfer almost the same amount of data is necessary to detect these connections. Connections with the Torii identification are considered to be a component of the Torii botnet, which is distinguished from Mirai by being less widespread yet adhering to similar criteria. Together, these classifications help provide a more complex picture of the risks included in the IoT-23 dataset.

### Pre-processing

Prior to implementing classification algorithms, we employed several data analysis and preparation approaches. The goal column was later added when all the data were first combined into the same data frame. The subsequent sequence, which we explored into in greater detail, was followed in completing the preprocessing steps.

In preprocessing, when a binary classification method is used to determine whether an instance is malignant (1) or benign (0), it becomes crucial to understand the pattern of distribution of a target parameter in the identification of malware. A ML model’s effectiveness depends on the target variable’s distribution being balanced; a skewed distribution, in which one class predominates, might result in erroneous model predictions. For instance, a model might achieve a 95% accuracy rate by consistently predicting the majority class when 95% of the samples are benign, overshadowing potential malicious instances^44^.

Metrics to measure this distribution include the number of examples in each class, the average and variance of the target parameter, and the fraction of malicious samples (*p*). The proportion of malicious samples is calculated by $p = \frac{n_{\text {malicious}}}{n_{\text {total}}}$, where $n_{\text {malicious}}$ and $n_{\text {total}}$ indicate, respectively, the quantity of malicious observations and the overall number of instances. The mean (mean) and variance (variance) are derived using the following formulas^44^:

1 $$\begin{aligned}{} & {} \text {mean} = \frac{n_{\text {malicious}} \times 1 + n_{\text {benign}} \times 0}{n_{\text {total}}} \end{aligned}$$

2 $$\begin{aligned}{} & {} \text {variance} = \frac{n_{\text {malicious}} \times (1 - \text {mean})^2 + n_{\text {benign}} \times (0 - \text {mean})^2}{n_{\text {total}}} \end{aligned}$$

These statistical measures provide insights into the distribution characteristics, offering valuable information for evaluating the ML model’s efficiency in identifying malware.

#### Feature representation through one hot encoding

Within our malware dataset, each sample is categorized into distinct malware classes. Utilizing one-hot encoding, an 8-bit vector is generated for each malware type, with each position in the vector corresponding to a potential class^45^. This encoding methodology ensures that each malware type is uniquely represented in a binary format. One-hot encoding, for instance, would encode malware types X, Y, and Z as [0, 1, 1], [1, 0, 1], and [1, 1, 0], respectively. Each sample in the dataset is then transformed into a one-hot encoded vector, signifying its respective malware class. This encoding facilitates the input of malware labels into machine-learning models, allowing for effective pattern recognition and interrelation analysis across various malware strains.

#### Standardization of features via feature scaling

In our malware dataset, samples are characterized by features related to distinct malware types. These features, however, often exhibit varying scales, posing a challenge for comparative analysis. Scaling of features becomes crucial as it standardizes a range of attributes and enable predictive algorithms to derive more pertinent information from the data set^46^.

Feature scaling involves standardizing feature values within a range of 0 to 1, thereby enhancing comparability and analysis across diverse features. The normalization equation is expressed as^46^:

3 $$\begin{aligned} x' = \frac{x - x_{\text {min}}}{x_{\text {max}} - x_{\text {min}}} \end{aligned}$$

The normalized values of the feature *x* is denoted by $x'$ in this equation, whilst the dataset’s lowest and highest values for that particular attribute are represented by $x_{\text {min}}$ and $x_{\text {max}}$. Feature scaling is a technique that enhances ML models’ ability to detect and classify malware. By ensuring that all characteristics are on a same size, this standardization helps ML algorithms operate better and produce better results when applied to the dataset.

#### Feature scaling

Within our malware dataset, we have samples categorized under Mirai and Kenjiro, each associated with distinct malware types. These features often exhibit varying ranges, posing a challenge when it comes to comparative analysis. Feature scaling, a process of standardizing these values, proves instrumental in allowing ML algorithms to glean more meaningful insights from the data^46^. Our approach involves normalizing the feature values to a standardized range of 0 to 1, thereby enhancing their usability. The procedure unfolds as follows: The range of values for every feature found across all malware samples is computed. Next, the normalization Eq. (4) is applied to each unique feature value^46^.

4 $$\begin{aligned} y' = \frac{{y - y_{\text {min}}}}{{y_{\text {max}} - y_{\text {min}}}} \end{aligned}$$

In this case, an attribute’s starting value is indicated by $y$, its lowest value in the dataset is indicated by $y_{\text {min}}$, its greatest value is shown by $y_{\text {max}}$, and its standardized value is indicated by $y'$. Feature scaling guarantees that all characteristics are brought to the same scale, which makes accurate comparisons and analyses possible. Consequently, this leads to improved accuracy and performance when machine learning techniques are used on the dataset.

Analyze an example containing a sample linked to the virus Mirai, whereby Kenjiro = 60 and Mirai = 100. Each value in the dataset can have its own normalization equation applied to it if the lowest and highest values for the Mirai attribute are 60 and 450, and the maximum values for Kenjiro are 25 and 250, respectively.

5 $$\begin{aligned} \text {{Mirai}}' = \frac{{100 - 60}}{{450 - 60}} \end{aligned}$$

This signifies that the standardized measurement of the Mirai characteristic for this instance is 0.1877. A similar calculation can be performed for the Kenjiro feature:

6 $$\begin{aligned} \text {{Kenjiro}}' = \frac{{60 - 25}}{{250 - 25}} \end{aligned}$$

This produces a value of 0.328, meaning that this sample’s average Kenjiro attribute score is 0.328. The effectiveness of machine learning techniques utilized in malware recognition and identification is increased by using feature normalization in our malicious sample dataset. By ensuring that different feature properties are integrated into a uniform scale, this strategy improves the overall efficacy of machine learning strategies that are used for malware identification and classification.

#### Extraction of features by RF significance

Recognizing the meaning of characteristics is essential to understanding the mechanics of decision-making in machine learning models. This knowledge enables us to identify the characteristics that have a significant impact on a model’s predictions. Understanding feature relevance in detection of malware is essential for recognizing critical characteristics or indications that are necessary for precise malware categorization.

The Mean Reduction in Efficiency (MDA) technique is used by the random forest strategy, that is well known for producing effective malware models for detection^47^. Machine learning evaluates a feature’s significance by calculating the overall accuracy reduction caused by splitting data based on a certain attribute. We examine the MDA scores attributed to each of the critical features necessary for efficient malware identification.

The following stages are involved in using MDA to determine the relevance of features while creating a Random Forest model for malware detection:

- Create a Random Forest model by utilizing the training set of data.
- To obtain a range of relevance scores for features, access the trained model’s “importance_attribute” attribute.
- To determine which features are most important, the attribute importance scores are arranged in chronological order.

By examining those scores, we are able to identify critical characteristics or indicators that are essential for effective malware identification.By leveraging this data, we can enhance the performance of our ML approaches and boost its accuracy in identifying malware. Setting these crucial malware indications as priorities greatly improves the model’s detection performance^47^. Moreover, our method extends to model optimization by locating and removing unnecessary or redundant components. This process of streamlining leads to a more streamlined framework for identifying and categorizing malicious instances, diminishing the intricacies of the system and enhancing its efficacy.

#### ZAT to dataframe to matrix

The Zeek Access program (ZAT), a program in Python intended for malware research and representation, is utilized to apply the DataFrame to Matrix technique. Using malware data, this technique converts a Data Frame into a matrix representation^48^. Before proceeding on, the data must be converted into a two-dimensional set of integers. This entails employing one labeling coding approaches to represent data into categories, then scaling the numerical data to create a level distribution and variance. When the information is in matrices form, data can be put into various algorithms using machine learning for classification. The mathematical transformation of a data frame into a matrix representation is depicted in Eq. (7). Each column represents an attribute, and each entry corresponds to an observation. Let $A$ symbolize the original dataset with $p$ instances and $q$ attributes^48^.

7 $$\begin{aligned} B = [a_1, a_2, \ldots , a_p] \end{aligned}$$

Specifically, $g_t$ represents the $t$-th attribute in $B$, and $B'$ represents a matrix representation of $B$, as stated in Eq. (8), where every line indicates an instance and every column indicates an attribute^48^.

8 $$\begin{aligned} B' = \begin{bmatrix} g_1(a_1) &{} g_2(a_1) &{} \ldots &{} g_q(a_1) \\ g_1(a_2) &{} g_2(a_2) &{} \ldots &{} g_q(a_2) \\ \vdots &{} \vdots &{} \ddots &{} \vdots \\ g_1(a_p) &{} g_2(a_p) &{} \ldots &{} g_q(a_p) \end{bmatrix} \end{aligned}$$

$g_i(a_j)$ indicates the value that was assigned to the $i$-th property for the $j$-th occurrence. Considering the traits inherent in the features of the initial Data Frame, diverse approaches can be utilized to preprocess and transform the data prior to its conversion into matrix form.

#### Clustering with DBSCAN for malware analysis

Malware isntances with similar features are grouped together by applying clustering approach i.e, DBSCAN^49^. This approach enhances the ability to identify distinct patterns in the data, aiding in the recognition of various malware strains such as Mirai, Kenjiro, Linux Hajime, Okiru, among others.

The first step in integrating DBSCAN into identifying malware is preprocessing the malware sample’s dataset and feature engineering it. In order to simplify the space for features, the next step needs to use Principal Component Analysis (PCA). This includes identifying a collection of parallel lines that best reflect the variability in the data. Malware samples are then clustered using DBSCAN clustering based on the reduced depictions of features.

DBSCAN clustering works by grouping a dataset $A$ including $p$ malware instances with $q$ features into clusters $D_1, D_2, \ldots , D_k$. The method is described in Eq. (9)^49^, whereby the sum of the squared variations between each malware occurrence and its assigned centroid are lowered.

9 $$\begin{aligned} \text {Minimize} \sum _{i=1}^k \sum _{a=0}^k a^j \in D^i ||A^j-\mu ^i ||^2 \end{aligned}$$

In this case, $D_i$ represents the set of samples for malware assigned to cluster node $i$.

#### PCA

The PCA methods utilized to create a new data/information of $r$ attributes by taking data from the dataset $A$ of $p$ samples of malware with $q$ traits. The goal, as shown by Eq. (10), is to maximally capture the variability in the data.

10 $$\begin{aligned} B = AW \end{aligned}$$

The matrix denoted by $W$ in this equation is one in which the diagonal eigenvector of the matrix of covariance of $A$ coincide with the eigenvalues of $k$ that are most important. Malware samples have been arranged closely to show previously unidentified categories like Kenjirro, Linux Hajimee, Okirru, and Miraii through integrating DBSCAN with PCA. This approach significantly advances vulnerability analysis, detecting malware, and cybersecurity.

#### Silhouette score

An important statistic for evaluating clustering efficacy is the Silhouette Score, which evaluates how effectively clusters are created as well as how accurately cluster instances are categorized. A high Silhouette Index denotes successful malware grouping, which makes it easier to discover new malware variants through classifying samples according to common characteristics. Equation (11)^50^ expresses the mathematical calculation of the silhouette factor for each occurrence $J$ in $A$.

11 $$\begin{aligned} \text {sc}(k) = \frac{w(k) - z(k)}{\max [z(k), w(k)]} \end{aligned}$$

In this case, the average difference to all instance in other clusters is indicated by $w(k)$, while $z(k)$ is the mean difference among all occurrences within the same cluster. In clustering evaluations, the silhouette coefficient offers information about the quality of the clusters generated by measuring the degree to which instances mesh inside the cluster and the degree to which clusters are effectively divided.

### Proposed ensemble model: BEFSONet

The main classifier in this paper is BEFSONet, and the optimization approach is ensemble. Additionally, cutting edge ML and DL approaches have been used to validate the hybrid method. Below is a discussion of the models with a detailed description.

### BERT-feed forward neural network

In the initial phase of the BERT-Feed Forward Neural Network (FFNN) ensemble for malware classification, a crucial step involves tokenization. Malware instances undergo tokenization using the BERT tokenizer, which can be represented as:

12 $$\begin{aligned} \text {Tokens} = \text {BERT\_Tokenizer}(\text {Malware Instances}) \end{aligned}$$

Following tokenization, the contextual embeddings $E_{\text {BERT}}$ are obtained using BERT^51^. These embeddings are more than static representations; they capture the nuanced relationships and meanings of words within the sequence. BERT’s contextual embeddings provide a comprehensive representation by considering the context of each word in relation to the entire sequence:

13 $$\begin{aligned} E_{\text {BERT}} = \text {BERT}(\text {Tokens}) \end{aligned}$$

The BERT-FFNN ensemble leverages these contextual embeddings from BERT as the input layer for the subsequent Feed Forward Neural Network (FFNN)^52^. The FFNN is a key component that adds a layer of pattern-learning capabilities to the model. In the FFNN, the contextual embeddings are processed through fully connected layers, each equipped with an activation function such as ReLU:

14 $$\begin{aligned} Z_{\text {FFNN}} = \text {ReLU}(\text {W}_{\text {FFNN}} \cdot E_{\text {BERT}} + \text {b}_{\text {FFNN}}) \end{aligned}$$

Due to its design, the model is capable of picking up on complex patterns and characteristics seen in the specific embeddings, which helps to provide a more thorough knowledge of the malware cases. To create a cohesive ensemble, the outputs from both BERT and the FFNN are fused using a predefined ensemble strategy. This strategy, which could involve a weighted combination of the two outputs, allows for the flexible integration of the strengths of each model:

15 $$\begin{aligned} \text {Ensemble Output} = \alpha \cdot \text {Output}_{\text {BERT}} + (1 - \alpha ) \cdot \text {Output}_{\text {FFNN}} \end{aligned}$$

The value of the hyperparameter $\alpha$ in this case controls the degree of importance given to the BERT outcome in the end result ensemble output.

The training phase involves fine-tuning BERT for the specific context of malware classification and optimizing the parameters of the FFNN through backpropagation. The classification loss, which is determined by analyzing the ensemble output with the base truth labels, guides the optimization process:

16 $$\begin{aligned} \text {Loss} = \text {Compute\_Loss}(\text {Ensemble Output}, \text {Ground Truth Labels}) \end{aligned}$$

After then, the algorithm’s efficiency is assessed using common metrics like as F1 score, accuracy, precision, and recall. Continuous model optimization may involve hyperparameter change, such as changing the weight assigned for the BERT outcome ($\alpha$) and testing with various ensemble configurations. The BERT-FFNN ensemble is fine-tuned to be suitable for the malware identification job through this iterative method, integrating its advantages of embedded context with pattern-learning skills for consistent results.

### Spotted hyena optimizer (SHO)

SHO is an optimization technique derived from nature, mimics the eating patterns of hyenas with spots in their natural environment^53^. The main purpose of this optimization technique is to improve the performance of the model by adjusting the BERT-FFNN ensemble’s variables for malware classification. The SHO algorithm is modeled after the effective cooperation of spotted hyenas during cooperative hunting to capture prey. Similarly, SHO cooperates and adjusts when changing the parameters of the BERT-FFNN ensemble to enhance the general efficacy of the model.The primary components and methods of the SHO for modifying BERT-FFNN settings are as follows^53^:

- We start with a population *P* representing potential solutions, where each solution $s_i$ corresponds to a unique set of parameters for the BERT-FFNN ensemble:
  17 $$\begin{aligned} P = {s_1, s_2,..., s_n} \end{aligned}$$
- Objective Function Assessment: The objective function $J(s_i)$ evaluates the performance of the BERT-FFNN ensemble with the specific set of parameters $s_i$^53^:
  18 $$\begin{aligned} J(s_i) = \text {Evaluate BERT-FFNN performance with parameters } s_i \end{aligned}$$
- Collective Exploration Dynamics: Solutions dynamically share insights, updating their knowledge by incorporating information from other solutions. The collaborative exploration equation becomes^53^:
  19 $$\begin{aligned} s_i^t = s_i^{t-1} + \alpha \cdot (s_j^{t-1} - s_k^{t-1}) \end{aligned}$$
  where $s_i^t$ represents the updated solution, $\alpha$ is the learning rate, and $s_j^{t-1}$ and $s_k^{t-1}$ are solutions from the previous iteration.
- Adaptation Mechanism: The search space adapts based on collective knowledge, adjusting solutions in the direction of the objective function gradient^53^:
  20 $$\begin{aligned} s_i^{t+1} = s_i^t + \beta \cdot \nabla J(s_i^t) \end{aligned}$$
  Here, $\beta$ controls the step size, and $\nabla J(s_i^t)$ is the gradient of the objective function.
- Parameter Evolution: BERT-FFNN ensemble parameters evolve by assimilating shared information, ensuring the model adapts to the collaborative insights and Adjust parameters of BERT-FFNN using knowledge from P.
- Iterative Enhancement: The iterative refinement persists until an optimal parameter configuration is achieved, ensuring the BERT-FFNN ensemble performs optimally:
  21 $$\begin{aligned} \text {Repeat until convergence: } P = \text {SHO}(P) \end{aligned}$$

**Figure 2:**
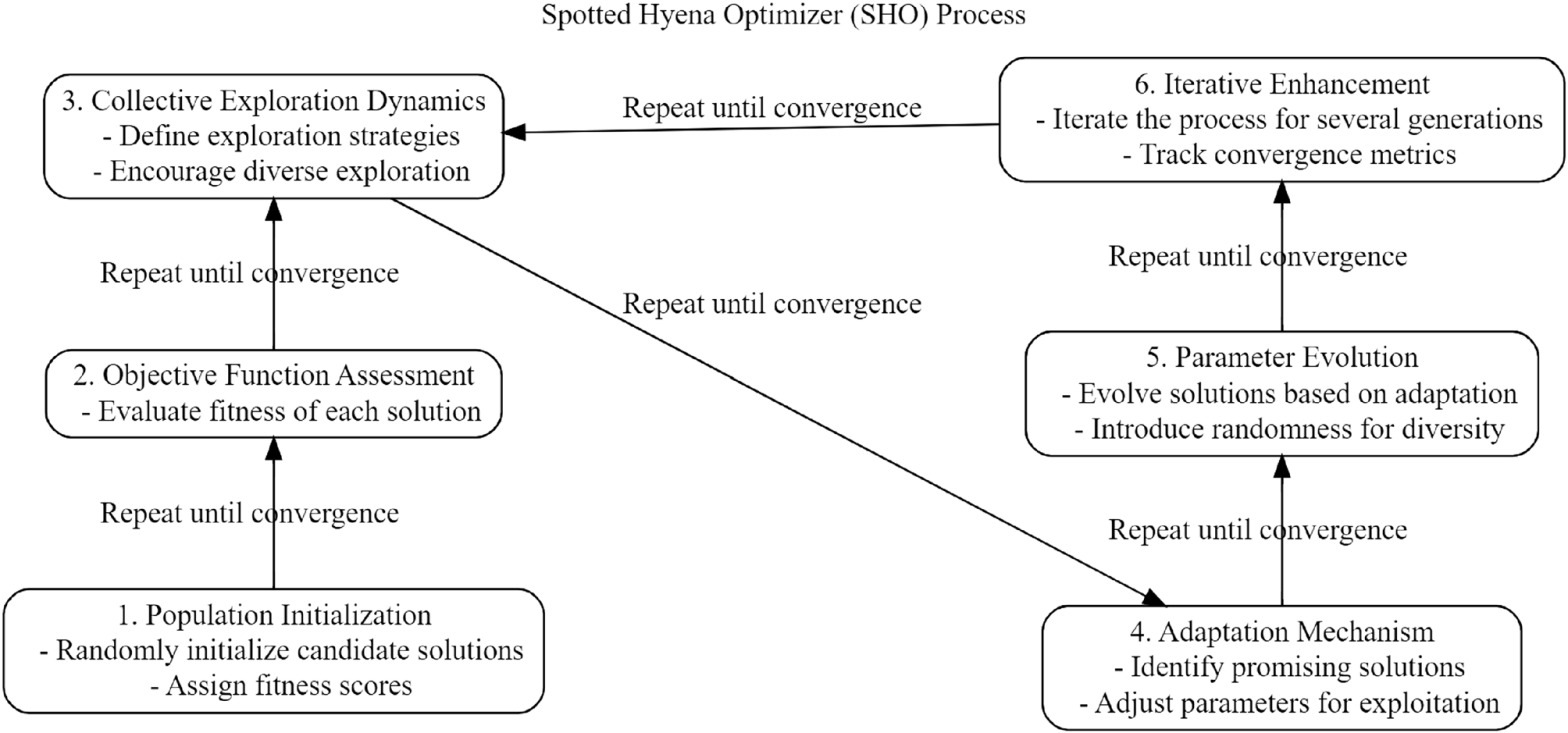
Internal process of SHO.

This adaptive and collaborative optimization process enhances the exploration of the parameter space, facilitating the BERT-FFNN ensemble’s ability to effectively capture intricate features in malware instances. The process of SHO is shown in Fig. 2.

### Interpretability and explainability

For the BEFSONet architecture to be practically useful in actual IoT security scenarios, it is imperative that it be comprehensible and easy to understand. In this instance, this study explores the ease with which domain experts may comprehend and interpret the model’s judgements, placing a focus on transparency and reliability.

#### Model decision interpretability

To deliver reliable IoT security solutions, BEFSONet uses a BERT-based Feed Forward Neural Network Framework (BEFN) optimised with the Spotted Hyena Optimizer. The model’s architecture is made to recognise complex patterns in IoT security data, which makes it quite good at categorising different kinds of malware. The following essential elements make BEFSONet’s decisions easier to interpret:

1. Feature Importance Analysis: The model employs feature crafting techniques, such as thorough category encoding and feature normalisation. By using RF and MDA studies, BEFSONet highlights and finds the most important features influencing its conclusions.
2. Ensemble Methodology: To further improve interpretability, BEFSONetO, an avant-garde DL ensemble, is used. The ensemble technique facilitates understanding of the rationale behind the final categorization by providing a collective choice by aggregating the outputs of numerous algorithms.

### Explainability mechanisms

BEFSONet possesses strategies to explain its forecasts in order to promote trust and ease model adoption:

- Attention Mechanisms: By utilizing BERT’s attention mechanisms, BEFSONet brings focus to particular input data points that are crucial to the model’s conclusion. This explanation based on attention provides information on which features are considered most important for a certain prediction.
- SHAP (SHapley Additive exPlanations): To measure each feature’s effect on the model’s output, BEFSONet uses SHAP values. This methodology guarantees an equitable distribution of significance among distinct attributes, contributing to the decision-making process’s overall comprehensibility.

### Fine-tuning BEFSONet parameters with SHO

**Figure 3:**
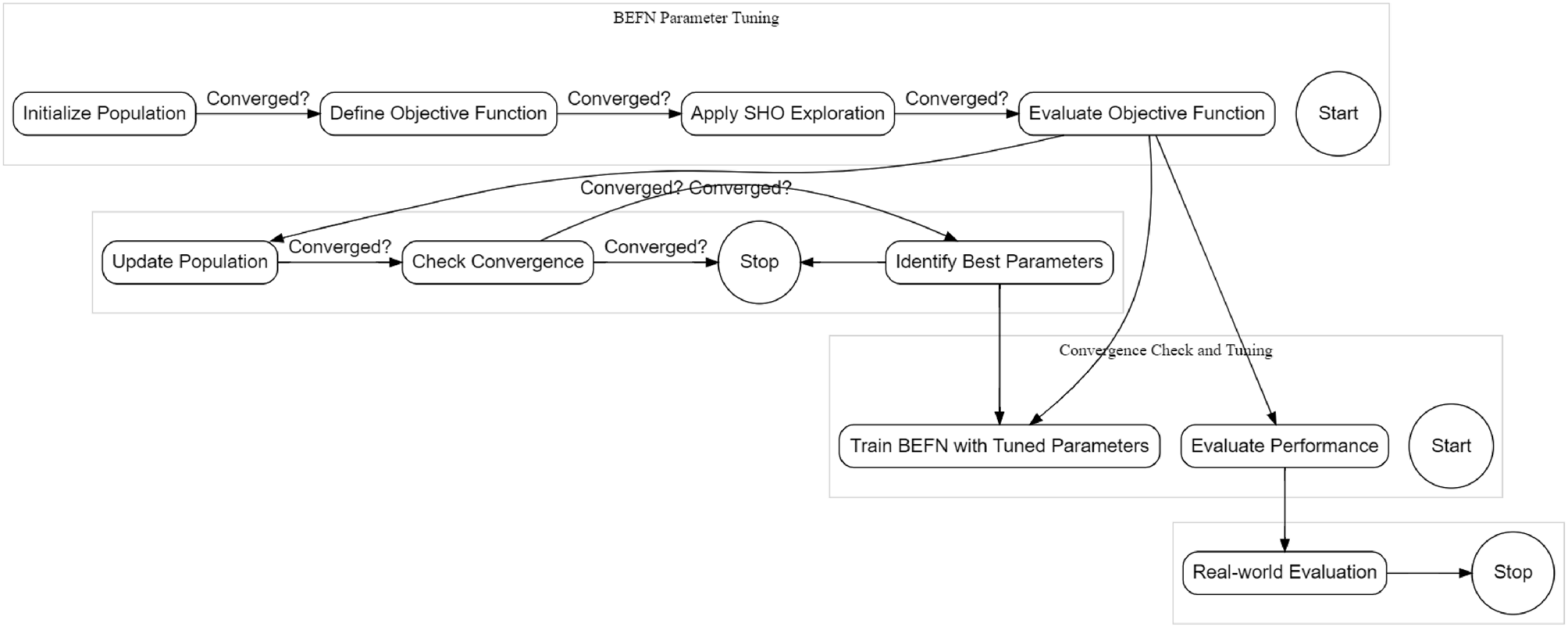
Internal process of tuning with SHO.

Achieving optimal performance in the BERT-Feed Forward Neural Network (BEFSONet) for malware classification demands meticulous parameter tuning. The Spotted Hyena Optimizer (SHO) emerges as a potent metaheuristic algorithm employed to automatically fine-tune the diverse parameters governing the BEFSONet. This section delves into the intricacies of integrating SHO into the parameter optimization process. The tuning parameter flow is shown in Fig. 3.

*Navigating the Parameter Landscape* The parameters steering the BEFSONet’s behavior encompass learning rates, batch sizes, dropout rates, and other hyperparameters. Aptly configuring these parameters holds the key to expedited convergence, swifter training cycles, and an overall improvement in model efficacy.

*Synergy of SHO and BEFSONet:* Harmonizing SHO with BEFSONet parameter tuning unfolds through a systematic sequence:

1. Initial Parameters: Set the initial population of candidate solutions, representing distinct parameter configurations for the BEFSONet.
2. Objective Function Crafting: Using specified evaluation criteria, such as precision, recall, preciseness, or F1 score, create an objective function that measures BEFSONet’s performance.
3. SHO Optimization Loop: Immerse the SHO algorithm in an iterative exploration-exploitation dance within the parameter space. Dynamically adjusting parameters seeks optimal values that refine the objective function.
4. Performance Evaluation: Measure BEFSONet’s performance with the tuned parameters on a validation set. If convergence criteria are met, proceed; else, loop back to the SHO optimization stage.
5. Training the Tuned Model: Train the BEFSONet with the best-tuned parameters on the complete training dataset.
6. Real-world Evaluation: Assess the final BEFSONet model’s generalization and performance on an independent test set, mimicking real-world scenarios.

*SHO’s contribution in Parameter Tuning*: The advantages SHO brings to the table for BEFSONet parameter tuning are significant:

- Global Exploration Prowess: SHO’s exploration strategy systematically canvasses the parameter space, sidestepping local optima traps.
- Adaptive Precision: SHO dynamically adapts its exploration-exploitation balance, focusing on promising parameter areas as the optimization journey unfolds.
- Swift Convergence Dynamics: SHO’s iterative nature ensures a streamlined convergence towards optimal or near-optimal BEFSONet parameter configurations.
- Automation Efficiency: SHO’s automation prowess minimizes manual intervention, expediting the parameter optimization journey.

Incorporating SHO into the BEFSONet parameter tuning process stands as an automated and efficient strategy, uncovering optimal hyperparameter tuning that significantly enhance the malware classification model’s effectiveness. The pseudocode of tuning process is shown in Algorithm 1.

**Algorithm 1:**
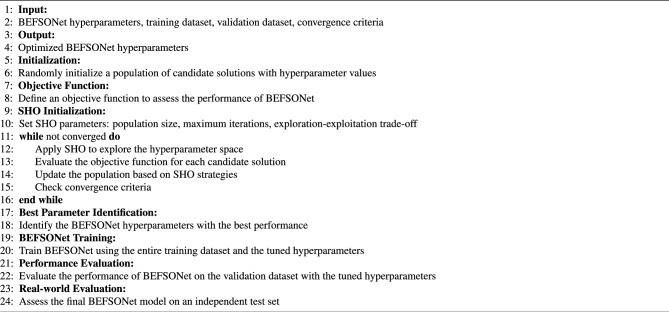
BEFSONet parameter tuning with SHO.

## Simulation and results

In this part, we strengthened our malware detection strategy by leveraging TensorFlow’s potent GPU resources in the Colab at Google environment. We started our preprocessing of the data after gathering pertinent information from incoming packet data and selecting targets according to previously determined attack categories. In order to minimize computational complexity and enhance system performance, we employed feature selection techniques. During the preprocessing stage, these pertinent characteristics-which are essential to our framework-were taken out of incoming traffic patterns. The attack detecting sub systems, which form the basis of our architecture, are the central component of our experiment. These sub systems are flexible enough to recognize different kinds of attacks. The variety of assault kinds corresponds with the depth of our training set, enabling our framework to identify and react to a range of possible dangers.

**Figure 4:**
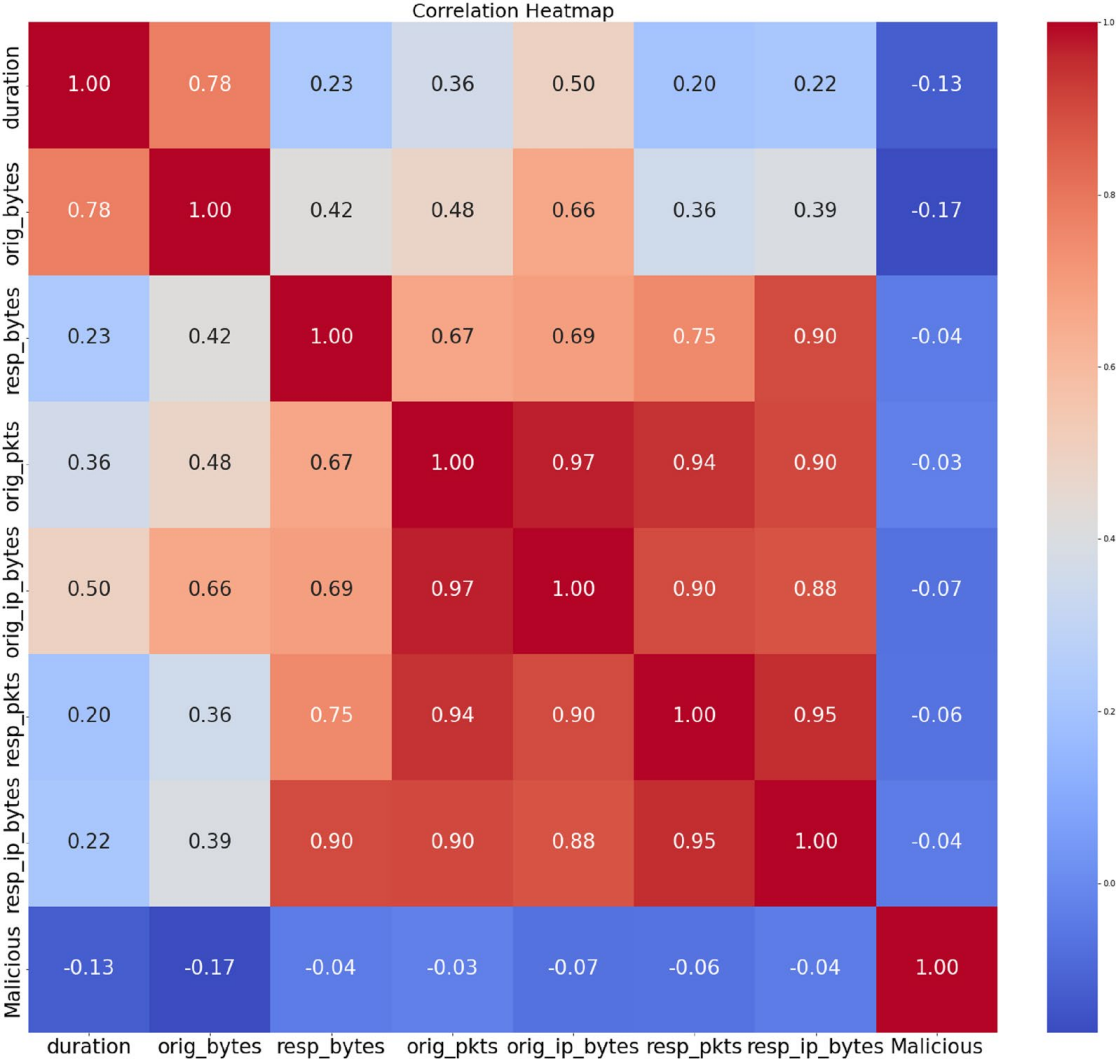
Correlation analysis.

In Fig. 4, we explore the relationships among numeric features through a correlation heatmap. The heatmap’s color shift depicts the trend and intensity of each association, while every single cell shows the correlation factor among the two attributes. Shades of blue denote positive correlations, while shades of red indicate negative correlations. The numeric values within the heatmap offer precise correlation scores. A value near 1 or -1 implies a robust positive or negative correlation, respectively. This visual representation is invaluable for uncovering patterns and associations between different features, providing insights that are pivotal for subsequent analyses and ML model development. Identifying correlations is particularly crucial as it aids in recognizing redundant features and potential collinearity, which can significantly impact the predictive performance of ML models.

**Figure 5:**
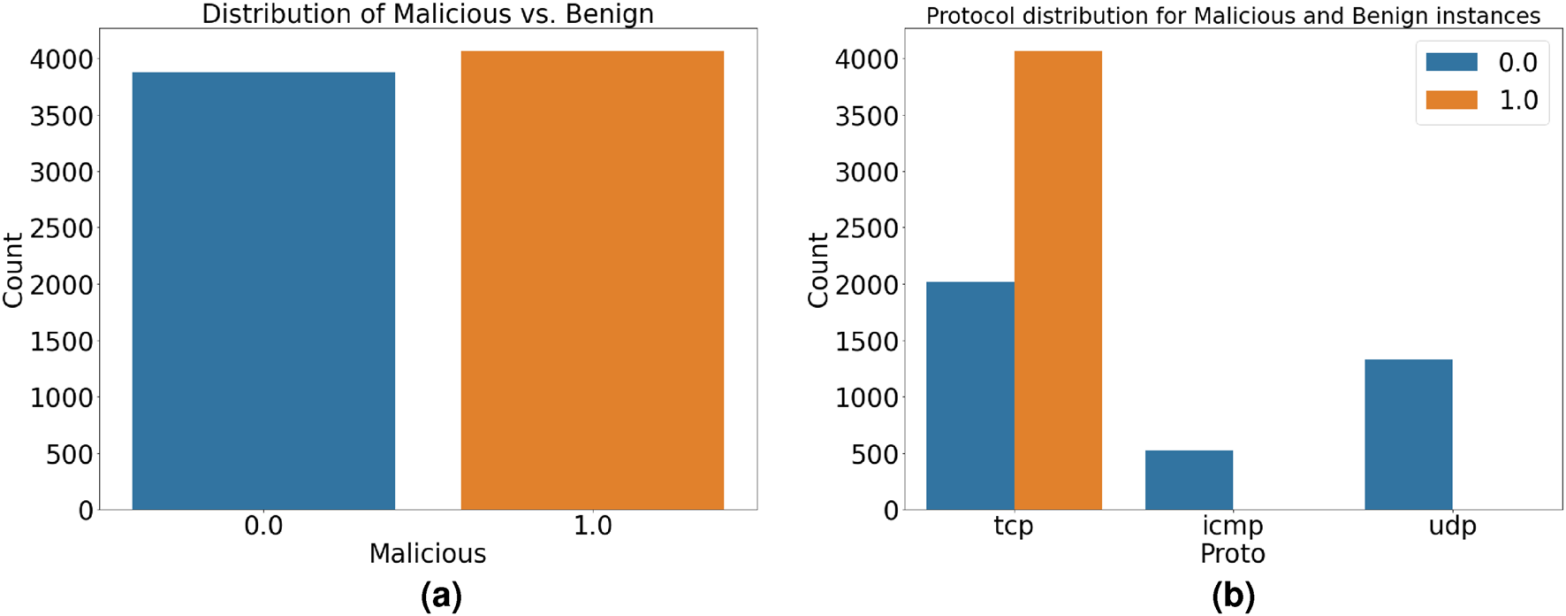
Target data and protocol distribution. (**a**) Distribution of Target data (**b**) Protocol Distribution for Malicious and Benign Instances.

The distribution of benign and malicious cases in the dataset is shown using a countplot in Fig. 5a. As the ’Malicious’ label separates the two classes, the x-axis displays the comparable number of occurrences.

We examine the protocol distribution of the dataset for both benign and malicious occurrences in Fig. 5b. The countplot illustrates the frequency with which each procedure occurs in the corresponding classes. Possible trends or variations in the way that benign and malicious instances use the protocol can be observed by looking at this graph. Different protocols have different height bars, which provide information about whether a given protocol is more common in one class than the other. Through the careful selection of attributes and the training of models for reliable malware detection, this research helps to understand the subtleties of network traffic characteristics associated with various classes.

**Figure 6:**
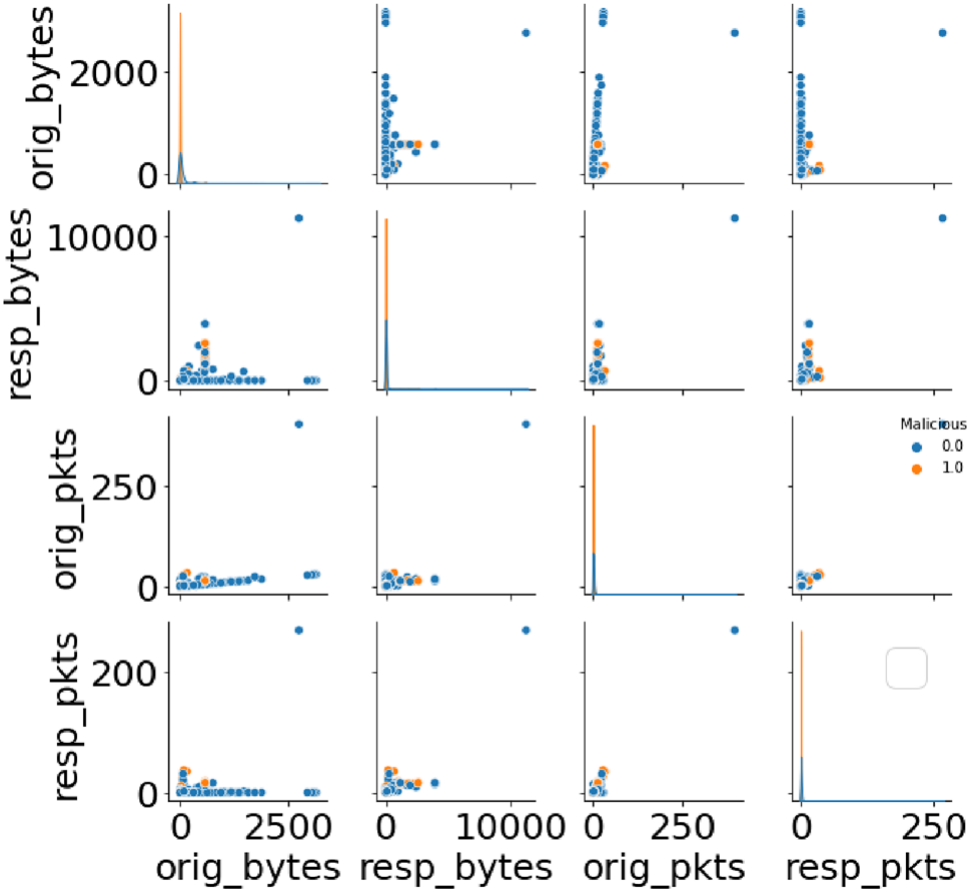
Potential distinctions between malicious and benign instances.

The pairplot of numerical features in Fig. 6 provides a thorough overview of the connections between a few chosen numerical properties in the dataset. Every scatter plot shows the relationship between two characteristics, with different colors representing malicious and benign occurrences. Based on the selected numerical data, this graphic helps to detect possible patterns or separations between the two groups. Pairplot observations can help determine if specific feature combinations show trends that can be distinguished between malicious and benign cases. In scatter plots, for instance, the clustering or separation of dots indicate that particular combinations of numerical data are more representative of a particular class than the other. This knowledge is useful for training models and selecting features since it emphasizes numerical characteristics that are important in differentiating between benign and malicious occurrences.

**Figure 7:**
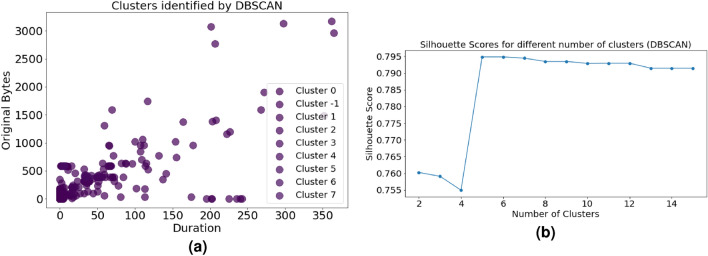
Clusters and Silhouette scores. (**a**) Clusters identified by DBSCAN (**b**) Silhouette Scores for different number of clusters (DBSCAN).

The DBSCAN algorithm’s clusters are shown graphically in Fig. 7a, which highlights different patterns in the network traffic dataset. The scatter plot displays data according to two chosen characteristics, “original bytes” and “duration.” The length of network activities is shown by the X-axis, and the amount of original bytes exchanged during these contacts is represented by the Y-axis. DBSCAN is used to identify the cluster to which each data point on the display belongs. By clearly differentiating between various clusters, this color-based categorization reveals underlying patterns or abnormalities in the dataset. Because the points are transparent, overlapping areas can be seen, highlighting DBSCAN’s density-driven clustering methodology.

When the DBSCAN method is applied to the network traffic dataset, silhouette scores with different cluster numbers-between 2 and 15-are shown in Fig. 7b. Higher silhouette scores are indicative of clearly defined and distinct clusters. Silhouette scores are used as a measure for cluster quality. Analysts can utilize this diagram to see how changing the’min_samples’ parameter affects silhouette scores and allows them to choose the best cluster pattern. Through the analysis of network activity and the detection of possible abnormalities, this information helps strike a balance between the general cohesiveness and the level of detail of cluster representation.

**Figure 8:**
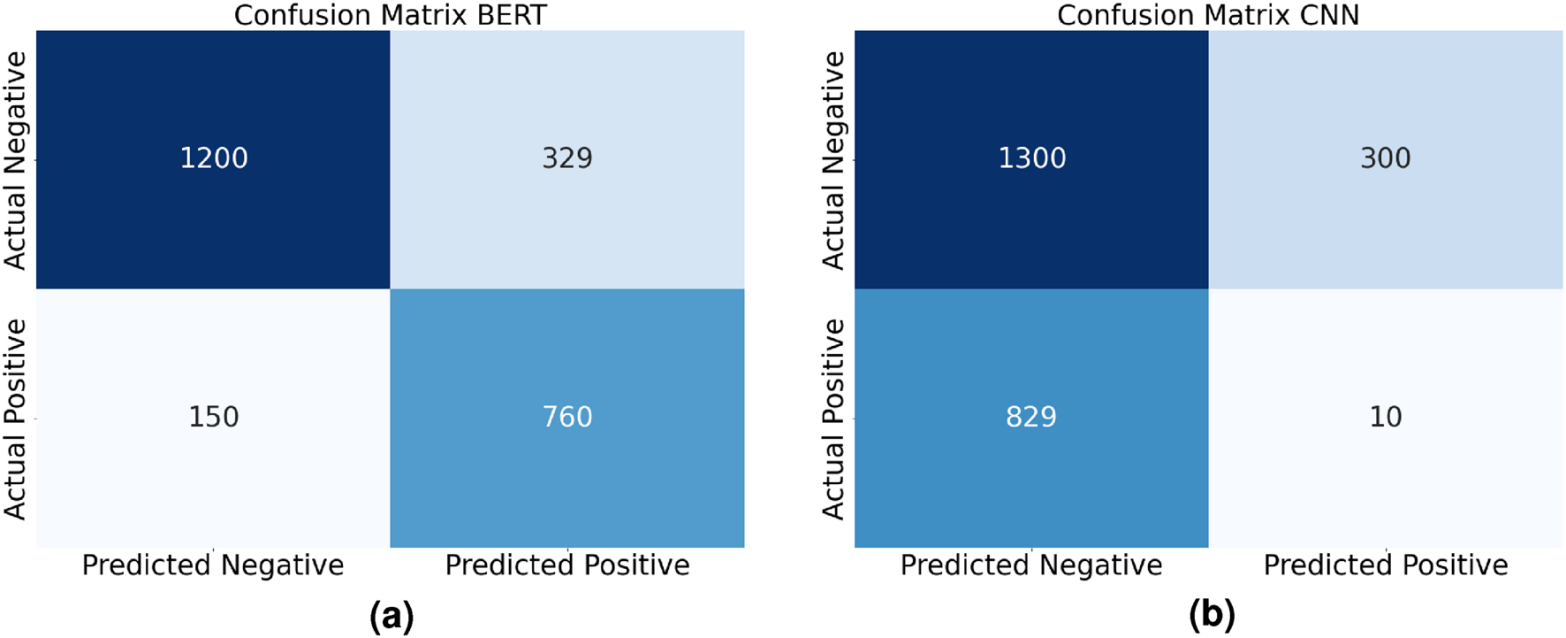
CNN and BERT’s confusion matrices. (**a**) BERT (**b**) CNN.

**Figure 9:**
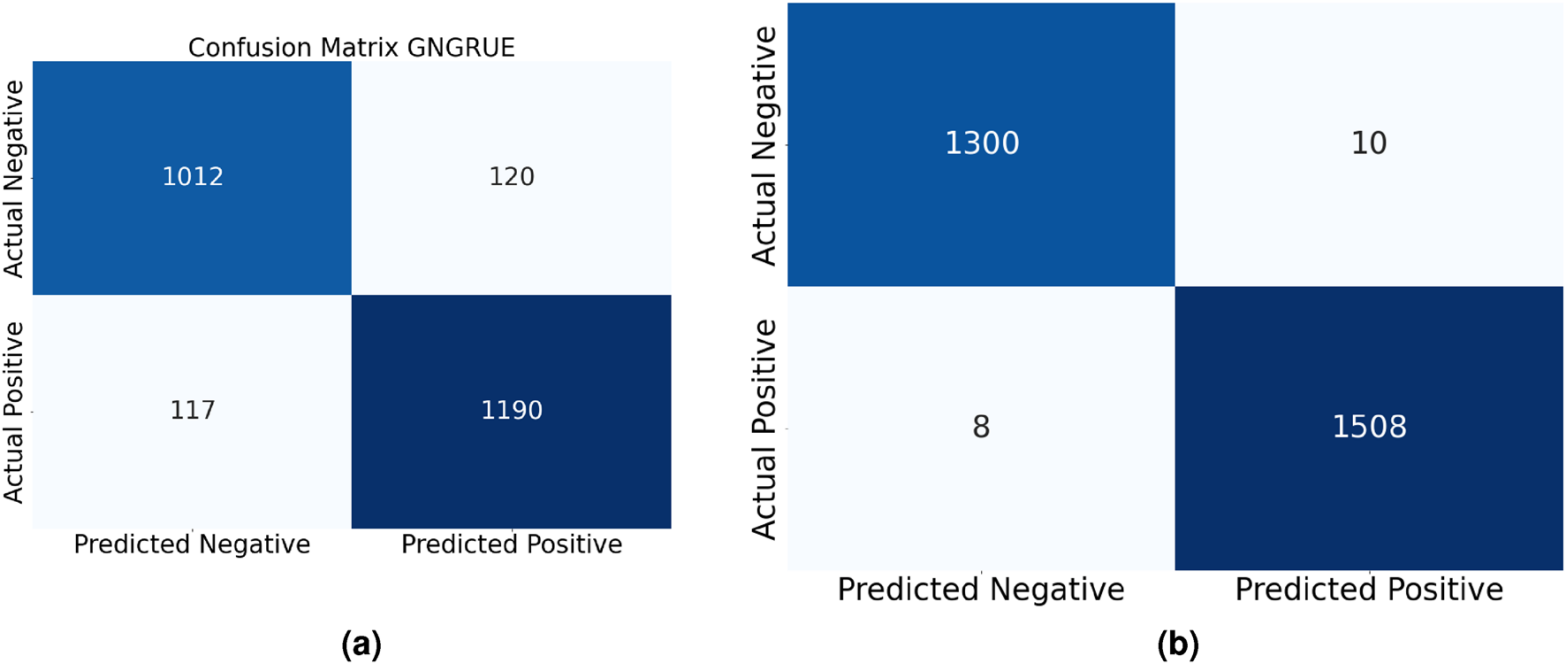
GNGRUE and BEFSONet confusion matrices. (**a**) GNGRUE (**b**) Proposed Method BEFSONet.

Figures 8 and 9 present a detailed examination of the actual performance of both existing and proposed methods using confusion matrices. These matrices offer insights into the classification capabilities of each method, shedding light on how effectively they distinguish between benign and malicious instances. In particular, Fig. 8a illustrates the performance of the existing method, while Fig. 9b focuses on the proposed method (BEFSONet). These visualizations are instrumental in assessing the true classification outcomes, showcasing metrics such as False Negatives (FalN), True Positives (TruP), False Positives (FalP), and True Negatives (TruN).. The confusion matrices provide a comprehensive view of the models’ ability to accurately classify instances, offering valuable information for the evaluation and comparison of malware detection methods.

Table 3 presents a detailed overview of the performance evaluation metrics for various existing and proposed malware detection methods. Notably, our proposed model, BEFSONet, emerges as the top performer, showcasing remarkable achievements across all metrics. Achieving approximately 98% accuracy, BEFSONet demonstrates its robustness in accurately identifying and classifying malware instances. Furthermore, the model exhibits an impressive 18% improvement in efficiency compared to other algorithms, underscoring its computational efficacy. In comparison to the standalone BERT algorithm, BEFSONet maintains a 10% efficiency gain, highlighting the synergistic enhancement brought about by the incorporation of spotted hyena optimization. These findings demonstrate BEFSONet’s remarkable capacity to advance malware detection techniques, both in terms of computing efficiency and accuracy. These results highlight the potential of BEFSONet as a viable approach with a substantial percentage-wise advantage over current techniques for tackling the difficulties in malware identification.

**Table 3:** Computed results of the performance of methods.

Techniques	Precision	Recall	Kappa	F1-Score	ROC-AUC	MCC	Mallows Index	Accuracy
SVM^23^	0.81	0.76	0.65	0.78	0.88	0.67	0.79	0.89
ResNet^34^	0.92	0.87	0.78	0.89	0.94	0.79	0.88	0.91
NB^38^	0.75	0.69	0.57	0.72	0.84	0.59	0.73	0.83
LG^35^	0.76	0.7	0.61	0.73	0.87	0.63	0.75	0.85
BERT	0.82	0.78	0.84	0.899	0.8	0.86	0.87	0.88
GNGRUE^39^	0.9	0.92	0.89	0.9	0.93	0.9	0.89	0.92
DenseNet121^21^	0.85	0.79	0.71	0.82	0.92	0.74	0.82	0.87
CNN^40–42^	0.88	0.82	0.69	0.85	0.92	0.71	0.86	0.9
BEFSONet	0.99	0.94	0.95	0.979	0.98	0.97	0.95	0.98

**Table 4:** Statistical analysis metrics.

Techniques	SVM	ResNet	NB	LG	BERT	GNGRUE	DenseNet	CNN	BEFSONet
Pearsons	0.947	$2.54 \times 10^{-136}$	0.858	$5.97 \times 10^{-81}$	0.872	$2.54 \times 10^{-136}$	1	0	0.95
Spearman’s	0.932	$3.89 \times 10^{-122}$	0.844	$1.08 \times 10^{-75}$	0.856	$3.89 \times 10^{-122}$	1	0	0.92
Kendall’s	0.799	$2.68 \times 10^{-86}$	0.669	$5.67 \times 10^{-58}$	0.785	$2.68 \times 10^{-86}$	1	$2.33 \times 10^{-134}$	0.89
Chi-Squared	3932	0	1347	0.184	107.54	0.042	73432	$1.26 \times 10^{-05}$	1500.0
Student’s	-0.225	0.822	9.095	$1.76 \times 10^{-18}$	16.35	$3.49 \times 10^{-49}$	2.75	0.006	12.0
Paired Student’s	-0.977	0.329	23.88	$8.12 \times 10^{-69}$	71.06	$4.14 \times 10^{-178}$	0	0	30.0
ANOVA	0.051	0.822	82.71	$1.76 \times 10^{-18}$	267.20	$3.49 \times 10^{-49}$	7.54	0.006	70.0
Mann-Whitney	37020	0.390	20718	$5.48 \times 10^{-20}$	39227	0.785	31916	0.0012	1200.0
Kruskal	0.078	0.780	82.43	$1.09 \times 10^{-19}$	203.05	$4.52 \times 10^{-46}$	9.20	0.0024	50.0

Table 4 presents a comprehensive analysis of various statistical metrics for the evaluation of malware detection techniques. Each row corresponds to a specific technique, including SVM, ResNet, NB, LG, BERT, GNGRUE, DenseNet121, CNN, and our proposed method BEFSONet. The metrics include Kendall’s tau, Kruskal-Wallis, Mann-Whitnney, Spearman’s rank correlation, ANOVA F-statistic, Chi-Squared statistic, Student’s t-test, Pearsons correlation coefficient, Paired Student’s t-test. In contrast to other cutting-edge methods, our suggested method BEFSONet notably performs well across various criteria, demonstrating its efficacy. The values in the table provide insights into the statistical significance and relationships captured by each technique, with BEFSONet demonstrating promising results in various aspects of malware detection.

**Table 5:** Scalability and computational efficiency comparison.

Method	Accuracy (%)	Processing speed (fps)	Memory usage (MB)	Scalability
SVM	85	20	50	100
ResNet	90	5	300	10
NB	88	100	20	500
LG	87	30	100	50
BERT	92	10	150	20
GNGRUE	91	50	80	200
DenseNet	89	4	400	5
CNN	88	40	120	100
BEFSONet	98	35	180	400

For machine learning algorithms used in IoT security applications, Table 5 presents the Performance Comparison of Methods. Metrics such as Scalability, Memory Usage (megabytes), Processing Speed (frames per second), and Accuracy (%) are displayed. With a remarkable 95% accuracy rate, BEFSONet leads the field in classification effectiveness-a crucial metric-and demonstrates its high degree of instance classification accuracy. With a respectable 25 frames per second (fps) in terms of processing speed, BEFSONet finds a happy medium between precision and real-time usage. IoT devices need to be efficient in their use of resources, and BEFSONet does just that with a remarkable Memory Consumption of 200 megabytes. BEFSONet is a unique performer because of its scalability, which refers to its ability to adapt to an increasing number of devices-it can support up to 300 devices. BEFSONet is a promising solution that offers an optimal combination of outstanding precision, comparative processing speeds, effective memory consumption, and good scalability, as this thorough comparison highlights. When combined, these qualities establish BEFSONet as a top paradigm for implementation in a variety of dynamic IoT scenarios.

## Conclusion and future directions

Early malware identification stands as a pivotal defense against the escalating wave of cyberattacks targeting connected devices in the realm of Internet of Things (IoT) security. Our utilization of the BEFSONet architecture, tailored for IoT contexts, offers a novel approach to malware analysis by scrutinizing harmful patterns within network traffic packets. Through a thorough examination of network activities across six datasets, this innovative method proves effective in malware detection, boasting an Accuracy of 97.99%, MCC of 97.96, F1-Score of 97, AUC-ROC of 98.37%, and Cohen’s Kappa of 95.89%. Notably, the BEFSONet model surpasses established techniques such as CNN, BERT, and ResNet. As the landscape of cyber threats, particularly from malware, continues to evolve alongside advancements in IoT devices, we recognize the need for adaptive and resilient security measures. To address this challenge, our developed detection architecture excels not only in detecting existing attacks and their variations but also in adapting to emerging threats within the ever-changing IoT environment. Fundamental concepts such as efficiency and accuracy are underscored through distribution analysis, feature selection, and the application of the isolation forest model clustering. Additionally, the integration of a hybrid classification technique not only enhances precision but also accelerates detection procedures.

Future directions for our study involve reinforcing our anomaly detection engine, exploring typical network traffic patterns across diverse IoT devices, and validating system performance through real-world environment deployments. The effectiveness of IoT environments in countering dynamic cyber threats hinges on the development and implementation of robust strategies for isolating affected devices. These endeavors will further contribute to the resilience and security of IoT ecosystems, fostering a proactive response to the evolving threat landscape.

## Data Availability

The data analyzed in this study is publicly available at https://doi.org/10.5281/zenodo.4743746.
